# Effects of N Reduction and Combined with Organic Fertilizer Application on Rice Yield and NH_3_ Volatilization from Saline Soil

**DOI:** 10.3390/plants15182814

**Published:** 2026-09-14

**Authors:** Jiayu Wu, Gui Chen, Yanfang Hao, Fan Tong, Xiaofang Zhao, Ying Chen, Shiwei Guo, Haijun Sun

**Affiliations:** 1 Co-Innovation Center for Sustainable Forestry in Southern China, College of Soil and Water Conservation, Nanjing Forestry University, Nanjing 210037, China; wujiayu24@njfu.edu.cn (J.W.); 18066042671@163.com (Y.C.); hjsun@njfu.edu.cn (H.S.); 2 Biotechnology Research Institute, Jiaxing Academy of Agricultural Science, Jiaxing 314016, China; 3 College of Hydraulic Engineering, Fujian Polytechnic of Water Conservancy and Electric Power, Yongan 366000, China; haoyanfang@hotmail.com (Y.H.); tongfanonline@163.com (F.T.); zhaoxiaofang2017@163.com (X.Z.); 4 Key Laboratory of Saline-Alkali Soil Improvement and Utilization (Coastal Saline-Alkali Lands), Ministry of Agriculture and Rural Affairs, Jiangsu Academy of Agricultural Sciences, Nanjing 210014, China; gsw@jaas.ac.cn

**Keywords:** ammonia volatilization, compost, nitrogen, rice production, urea

## Abstract

Organic fertilizer (OF) application can significantly enhance soil fertility and crop yield. However, its influence on nitrogen (N) loss via ammonia (NH_3_) volatilization from reclaimed coastal paddy soil is not well known. We conducted a pot experiment here using cyanobacteria compost of sawdust and chicken manure plus cyanobacterial sludge, with four treatments, i.e., no urea-N (CK), urea-N (U), 80% urea-N (RU), and 80% urea-N plus compost (RU+OF), to examine these effects. The results showed that reducing urea-N input increased rice grain yield by 29.9–52.1% compared to U treatment. However, RU+OF produced significantly lower rice grain than RU by 14.6%. More straw biomass and N uptake did not translate into higher rice grain yield under OF-amended treatment. Interestingly, compared to the other treatments, RU+OF significantly mitigated NH_3_ volatilization by 8.9–25.3%, achieving the lowest emissions and the greatest reduction. A 20% reduction in N application rate combined with compost enhanced crop apparent N recovery efficiency and lowered NH_3_ volatilization. Therefore, organic substitution through combined compost application has potential as a component of reduced-N fertilization strategies in reclaimed coastal paddy soil.

## 1. Introduction

Coastal regions suffer from cultivated land scarcity, so coastal saline-alkali soils have become important reserve resources for agricultural development [1]. Affected by seawater intrusion and human activities, these soils typically exhibit high pH, salinity, and electrical conductivity (EC) [2,3,4]. These conditions strongly restrict plant growth and microbial activity, and slow down soil nutrient transformation, thereby hindering the sustainable development of coastal agriculture in China [5,6]. However, following long-term reclamation and cultivation, nutrient management rather than salinity has become a major factor limiting crop productivity and increasing nitrogen (N) loss in reclaimed coastal paddy soils. Organic fertilizers can reduce soil salinity and alkalinity, boost soil nutrients, promote nutrient uptake by crops and improve crop yields [7]. Consequently, organic fertilizer substitution is a widely applied practice in rice production in saline-alkali soils worldwide [8].

Partial substitution of synthetic N with organic fertilizer can reduce mineral N inputs and improve soil N availability [9,10]. However, maintaining rice productivity while reducing N input requires an appropriate balance among crop N demand, fertilizer N supply, and potential N losses. Organic fertilizer application may also cause ammonia (NH_3_) volatilization, which accounts for 10–38% of annual N loss from farmland [11]. NH_3_ volatilization is a major pathway of N loss that not only lowers N use efficiency (NUE) [12,13], but also contributes to eutrophication and atmospheric N deposition. NH_3_ volatilization generally increases with fertilizer N application rate because greater N inputs elevate ammonium (NH_4_^+^-N) concentrations in floodwater and topsoil [14]. Therefore, the effects of reduced mineral N combined with organic fertilizer on crop production and NH_3_ volatilization should be evaluated simultaneously. Appropriate fertilizer types and substitution ratios, together with effective NH_3_ mitigation, are essential for maintaining crop yield and environmental sustainability [15,16].

Feedstock is another key factor determining the effects of organic fertilizer. Organic fertilizers derived from different feedstocks (e.g., livestock manure, straw, and green manure) vary in nutrients and physicochemical properties. This also applies to organic fertilizer amended with cyanobacterial sludge [17], which may exert distinct effects on soil improvement and NH_3_ volatilization. Additionally, organic fertilizer substitution ratios can affect soil fertility and crop yield [18]. Previous research indicated that sole organic fertilization or an excessively high substitution ratio may lower rice yield by 10.4% relative to conventional fertilization [19]. The compost used in the present study was selected based on our previous composting experiment, in which cyanobacterial sludge replaced 30% of the chicken manure and showed satisfactory maturity, nutrient quality, and resource-recycling potential. However, favorable performance during composting does not necessarily indicate comparable agronomic and environmental effects after soil application.

At present, limited information is available on the performance of this cyanobacterial-sludge-amended compost combined with reduced urea-N input in rice production. In particular, its integrated effects on NH_3_ volatilization, rice N uptake, apparent N recovery efficiency (ANRE), biomass allocation, and grain yield have not been evaluated concurrently. Therefore, the key question of this study was whether combining this compost with reduce N could simultaneously maintain rice productivity and reduce N losses.

To fill these research gaps, a pot experiment was conducted to examine the effects of N reduction combined with organic fertilizer application on rice yield, ANRE and NH_3_ volatilization from a reclaimed coastal paddy soil. This study aimed to identify a relatively optimal application strategy and support the practical use and environmental evaluation of such organic fertilizers. Accordingly, we hypothesized that reduced-N application combined with cyanobacterial-sludge-amended compost would maintain rice yield while reducing NH_3_ volatilization compared with reduced-N application alone.

## 2. Results

### 2.1. Rice Growth and Yield

**Figure 1:**
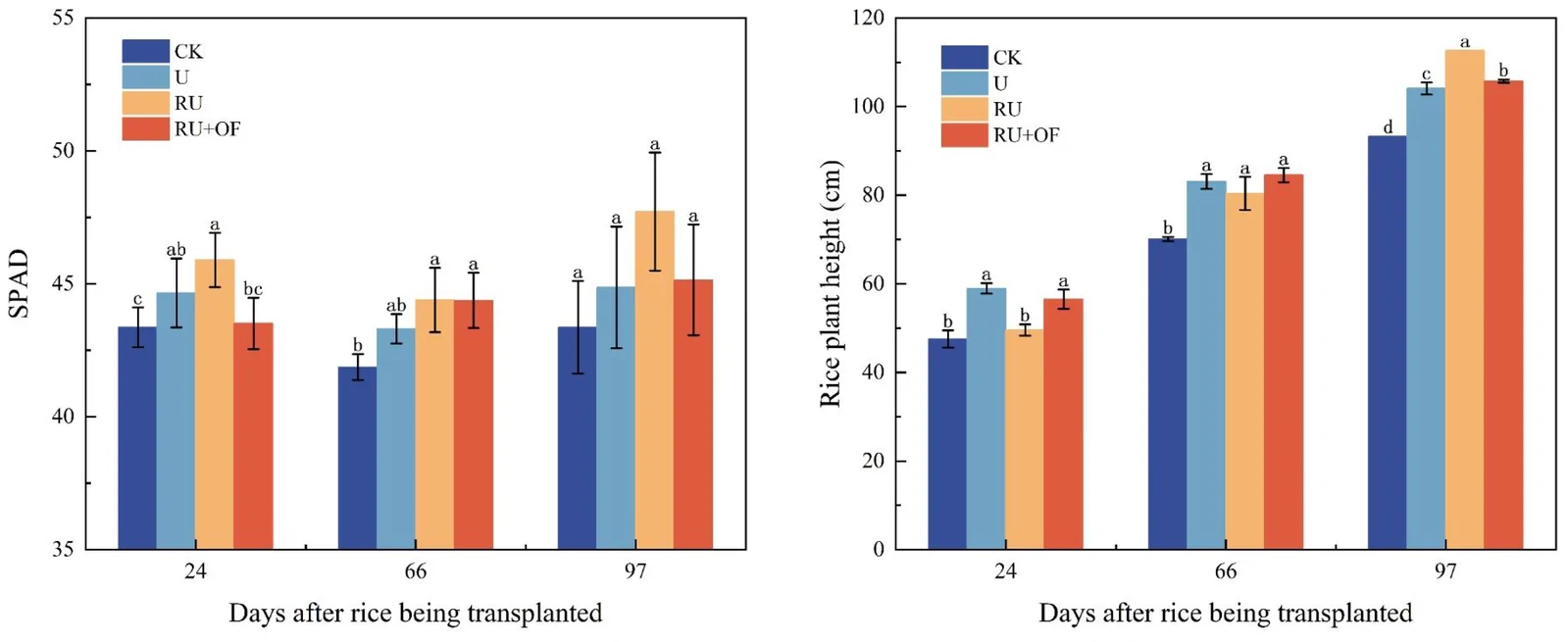
Changes in flag leaf SPAD and plant height of rice at different growth stages. Data were presented as means ± SD (*n* = 3). Different lowercase letters above the column indicate significant differences among treatments at *p* < 0.05.

During the observation conducted on the 24th day after rice transplantation, both U and RU treatments had 3.0–5.8% higher leaf SPAD values than CK treatment (Figure 1). Meanwhile, RU+OF treatment had a significantly lower leaf SPAD value than RU treatment, but this effect was overturned during the subsequent two observations on the 66th and 97th days after the rice seedlings were transplanted. On the second observation, rice plants under RU and RU+OF treatments had the highest leaf SPAD values of 44.38 and 44.39, respectively, which were 5.9% and 6.0% higher than those under CK treatment. At the third observation, there were no significant differences in flag leaf SPAD values among the treatments (Figure 1).

Plant height increased rapidly from the tillering to the jointing stage and gradually stabilized at the heading stage during the rice period (Figure 1). The three urea-N treatments increased plant height compared with the CK treatment. At the tillering stage, U treatment exhibited the highest plant height of 59.0 cm, exceeding the values under RU and RU+OF treatments by 8.4–19.0%. Notably, plant height of RU+OF treatment was close to U treatment and 9.8% higher than that of RU treatment. At the jointing stage, there was no significant difference in plant height among the N-fertilized treatments, although all were greater than that under CK treatment. The early advantage of U treatment in plant height was no longer observed at the jointing stage. At this stage, plant height under RU and RU+OF was 1.6–8.6% higher than that under U, and was 6.5% higher under RU than under RU+OF treatment, although the differences among the N-fertilized treatments were not significant (Figure 1).

Both reduced-N treatments increased rice biomass and yield-related indices compared with U treatment (Table 1). The straw biomass under RU+OF treatment was nearly 1.5 times that under RU treatment. Compared with U treatment, RU and RU+OF treatments significantly increased grain yield by 29.9–52.1%. Specifically, RU treatment had the highest grain yield (133.7 g pot^−1^), exceeding RU+OF treatment by 17.1%. The aboveground biomass under RU+OF treatment was the highest and was significantly higher than that under the RU treatment by 14.0%. There were no significant differences in 1000-grain weight among all treatments or in effective panicle number among the N-fertilized treatments (Table 1).

### 2.2. N Uptake, ANRE and N Harvest Index (NHI) of Rice

Reduced N combined with compost enhanced N uptake in rice straw and grain, as well as the ANRE (Table 2). Both RU and RU+OF treatments had higher grain N uptake and total N uptake than U treatment. RU+OF treatment produced the highest straw N uptake (1.2 g pot^−1^), which was 38.8% higher than that in RU treatment. Grain N uptake under RU+OF treatment was nearly twice that of U treatment, and increased significantly by 22.3% compared with RU treatment. RU+OF also showed the highest total N uptake (2.3 g pot^−1^) and ANRE (91.4%), with both values significantly higher than those under RU. In contrast, no significant differences in NHI were observed among the N-fertilized treatments, although RU and RU+OF showed numerically higher NHI values (52.4% and 49.4%, respectively) than U (35.0%).

### 2.3. NH_3_ Volatilization

**Figure 2:**
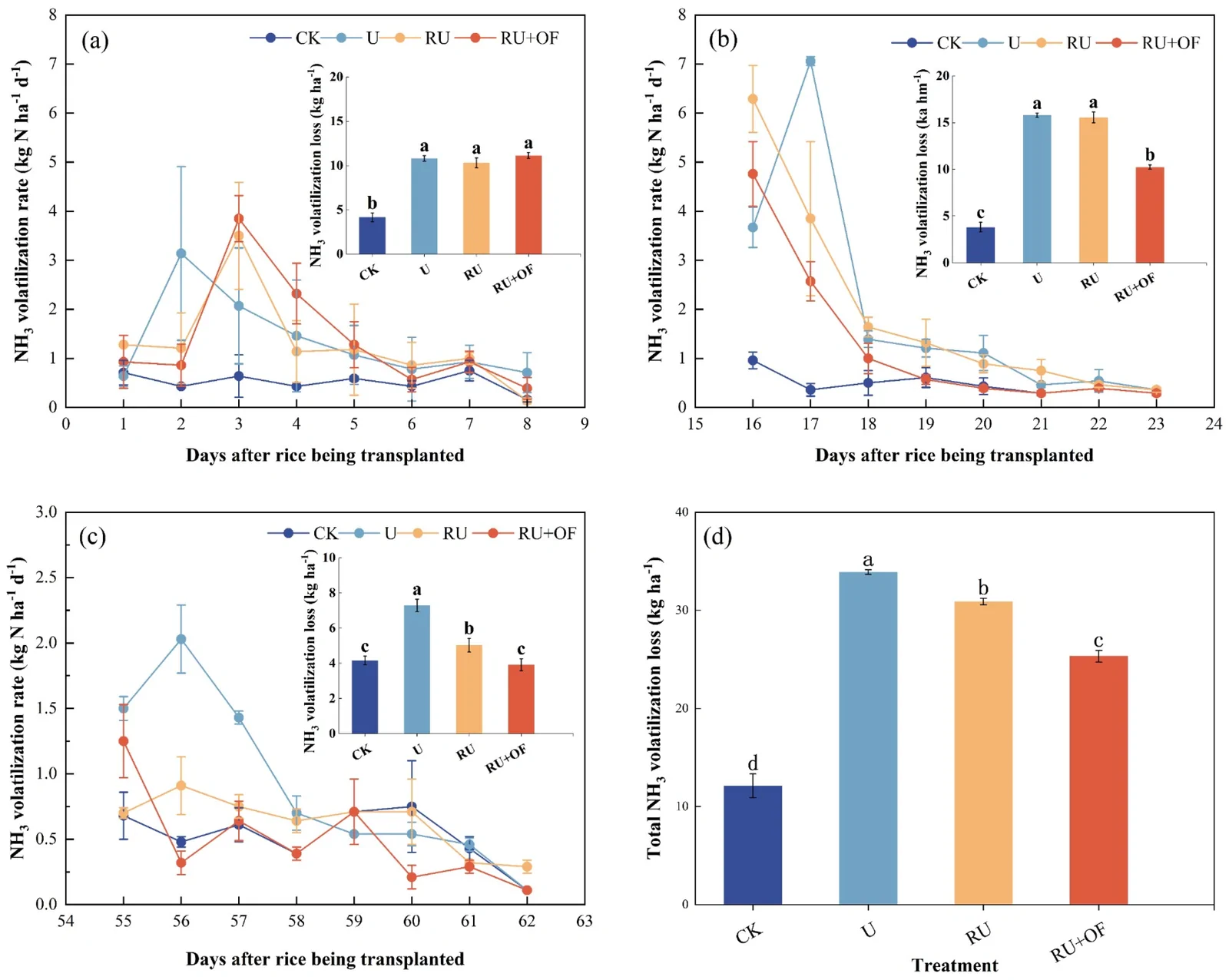
Effects of N reduction combined with organic fertilizer application on the ammonia (NH_3_) volatilization rate during the basal (**a**), tillering (**b**), and panicle (**c**) fertilization observations, as well as the total NH_3_ volatilization loss (**d**). Data are presented as means ± SD (*n* = 3). Different lowercase letters above the column indicate significant differences among treatments at *p* < 0.05.

The NH_3_ volatilization differed markedly among the N-fertilized treatments, while all treatments shared a comparable overall trend driven by N application (Figure 2). During the basal fertilization stage, the NH_3_ volatilization rate under each treatment peaked 2–3 days after N application, followed by a rapid decline, and approached the level of the CK treatment by day 5. RU+OF treatment reached a peak flux of 3.85 kg ha^−1^ d^−1^ on day 3. Cumulative NH_3_ volatilization was significantly higher in all N treatments than in CK, with no significant differences among them (Figure 2a). During the tillering fertilization stage, the NH_3_ volatilization rate of U treatment peaked on day 17 after transplanting (7.06 kg ha^−1^ d^−1^), whereas those under the other N-fertilized treatments peaked on day 16. Within three days after day 16, the differences among treatments narrowed, and all rates gradually approached the CK treatment level. Notably, RU+OF treatment generally maintained a lower NH_3_ volatilization rate than RU treatment. The cumulative NH_3_ volatilization in this period was markedly reduced by 35.1% and 34.1% in RU+OF relative to U and RU, respectively (Figure 2b). During the panicle fertilization stage, NH_3_ volatilization varied considerably among treatments. The U treatment showed the highest peak flux (1.71 kg ha^−1^ d^−1^), which was 1.8 times that of RU+OF treatment. Compared with the U treatment, RU and RU+OF had lower cumulative NH_3_ volatilization, and RU+OF was 22.1% lower than RU (Figure 2c).

Total NH_3_ volatilization mainly occurred during the basal and tillering fertilization stages, with the tillering stage accounting for 46.6% of total NH_3_ emissions (Figure 2d). The U treatment had the highest cumulative NH_3_ volatilization, reaching 33.90 kg ha^−1^. Compared with U treatment, the two 20% N reduction treatments significantly decreased cumulative NH_3_ volatilization by 12.8% and 25.3%, respectively. RU+OF treatment showed the lowest total NH_3_ loss (25.31 kg ha^−1^), which was 14.3% lower than RU treatment. The basal fertilization stage contributed the largest proportion of NH_3_ loss under RU, whereas the tillering fertilization stage contributed the largest proportion under RU+OF. Cumulative NH_3_ volatilization followed the order U > RU > RU+OF among the N-fertilized treatments (Figure 2d).

### 2.4. Floodwater pH, NH_4_^+^-N and Nitrate (NO_3_^−^-N)

**Figure 3:**
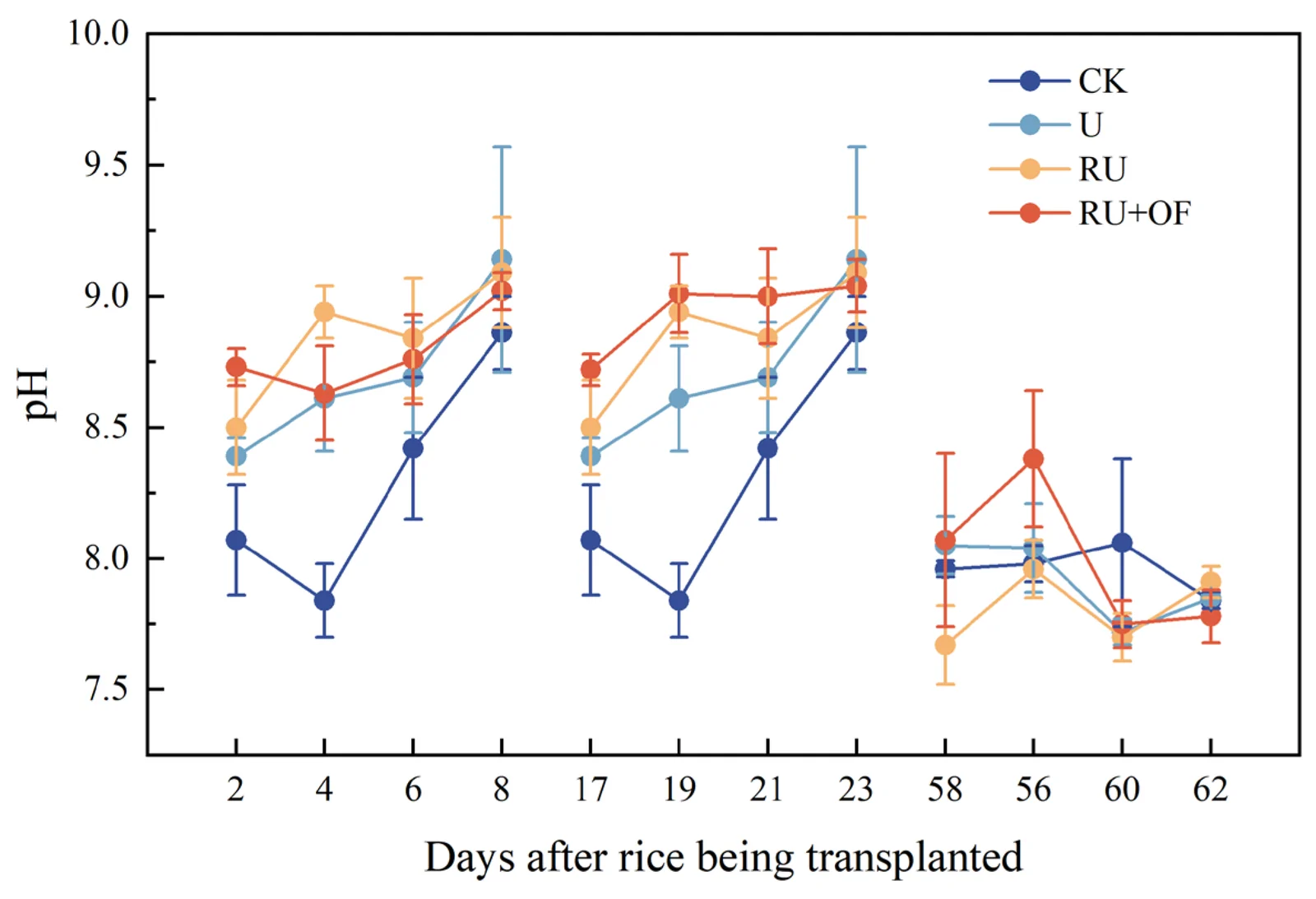
Changes in pH of floodwater in rice fields under different treatments during three N fertilization stages. Data were presented as means ± SD (*n* = 3).

The pH of floodwater in each treatment showed an overall increasing trend during the basal and tillering fertilization stages, but decreased during the panicle fertilization stage (Figure 3). Floodwater pH declined shortly after both topdressing events. Under U treatment, the floodwater pH initially decreased and then increased during the basal fertilization stage and the early period following panicle fertilization stage. By the end of the monitoring period, the pH values of all treatments stabilized at 7.67–8.07.

**Figure 4:**
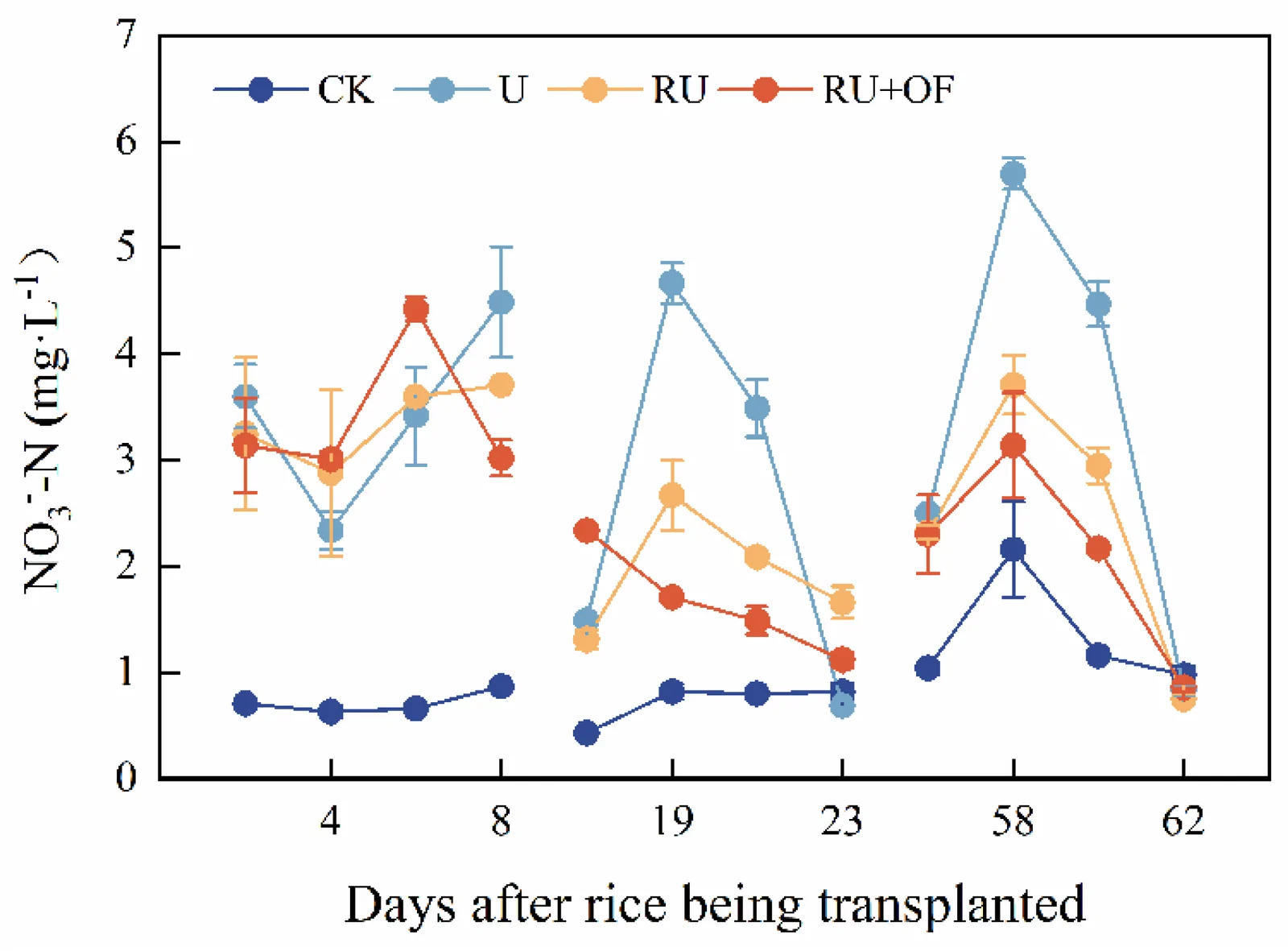
Changes in NO_3_^−^-N concentration in floodwater of rice fields under different treatments during three N fertilization stages. Data were presented as means ± SD (*n* = 3).

The average NO_3_^−^-N concentrations in floodwater across the three fertilization stages are presented in Figure 4. After basal fertilization, all treatments exhibited a similar trend, decreasing first and then increasing within the first six days. During this stage, no significant difference was observed between RU and RU+OF treatment, although RU treatment showed slightly lower concentrations during the early stage and higher concentrations thereafter. Across the monitoring periods, NO_3_^−^-N concentrations under RU and RU+OF treatments were generally lower than those under U treatment (average: 3.46 mg L^−1^), and this pattern persisted throughout the entire experiment. Compared with U treatment, RU treatment consistently reduced floodwater NO_3_^−^-N concentrations, especially during the tillering and panicle fertilization stages. The U treatment peaked at 5.70 mg L^−1^ on day 58. On day 62, the NO_3_^−^-N concentration in RU+OF treatment was 13.6% lower than that in RU and 59.5% lower than that in U treatment (3.38 mg L^−1^), with these differences being statistically significant (Figure 4).

**Figure 5:**
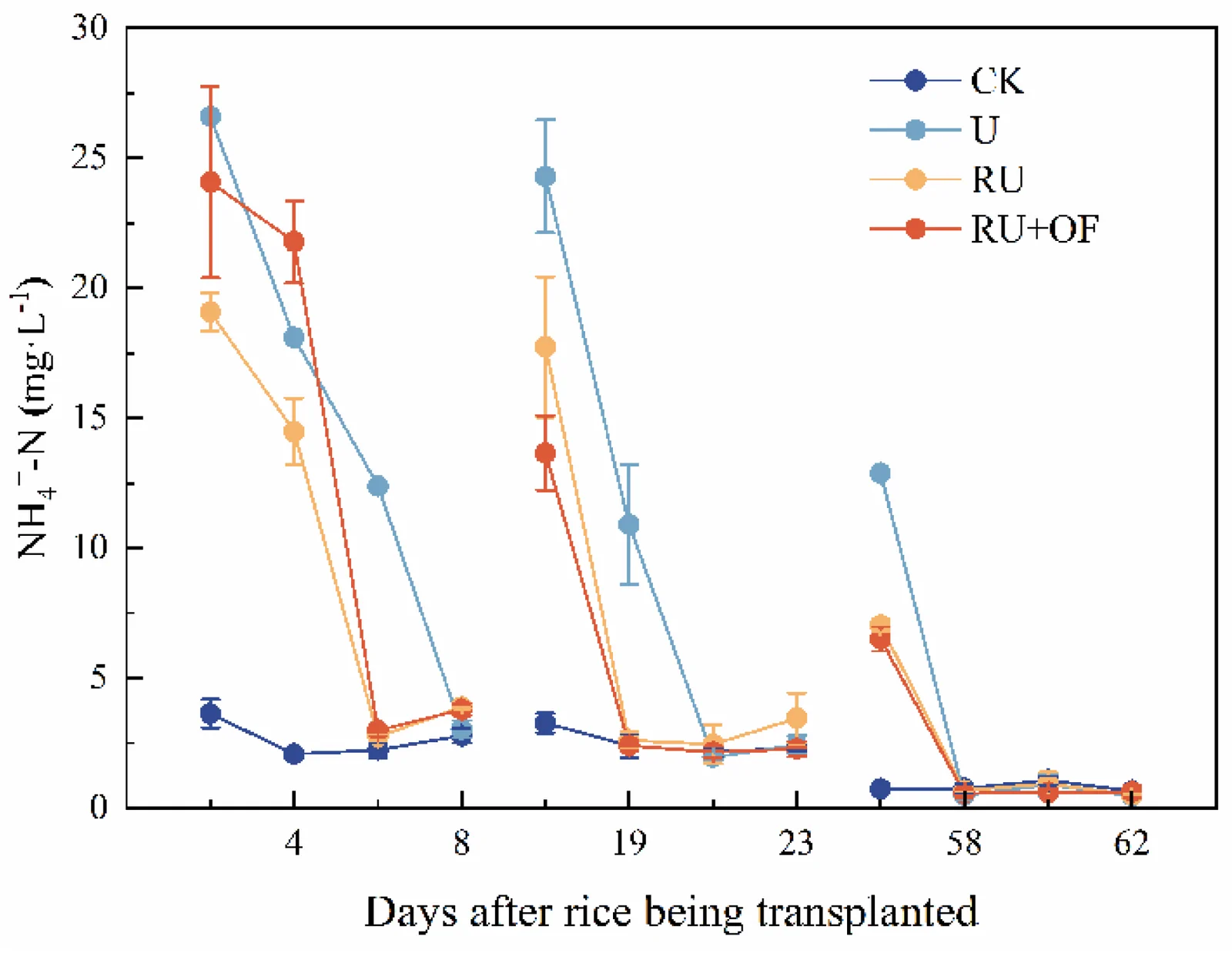
Changes in NH_4_^+^-N concentration in floodwater of rice fields under different treatments during three N fertilization stages. Data were presented as means ± SD (*n* = 3).

During the basal fertilization stage, floodwater NH_4_^+^-N concentration increased rapidly after N application and peaked on day 2 (Figure 5). The U treatment had the highest value of 26.6 mg L^−1^, after which the concentrations declined rapidly and stabilized after day 6. Compared with U treatment, RU and RU+OF treatments had markedly lower floodwater NH_4_^+^-N concentration. At the tillering fertilization stage, NH_4_^+^-N under all treatments also peaked on day 2. The concentration under U treatment was 37.1–78.0% higher than those under RU and RU+OF treatments. Notably, RU+OF treatment had a higher NH_4_^+^-N concentration than RU treatment at the tillering stage, but the trend reversed later, with a variation pattern similar to NO_3_^−^-N. The concentrations under all treatments except U treatment stabilized at 2.4–3.0 mg L^−1^ by day 4, while U treatment stabilized later at 1.90–2.05 mg L^−1^ by day 6. Across tillering and panicle fertilization stages, the concentration of NH_4_^+^-N in floodwater generally followed the order U > RU > RU+OF > CK (Figure 5).

## 3. Discussion

### 3.1. Effects of N Reduction and Combined with Compost on Rice Production

The present study showed that reduced-N regimes maintained or improved rice growth and grain yield relative to full-rate N application treatment [20,21]. Previous studies have reported that partial organic fertilizer substitution can maintain rice yield by improving the source–sink balance during grain filling [22,23]. Our results further indicate that adding cyanobacterial-sludge-amended compost under a 20% urea-N reduction altered biomass allocation and yield formation, although the effects of N reduction and compost addition could not be statistically separated in the present experimental design.

In terms of grain yield, both N-reduced treatments achieved significantly higher yields than U treatment. RU treatment had the highest grain number per panicle and harvest index, suggesting efficient conversion of accumulated biomass into grain. Because RU received reduced urea-N without compost, this response cannot be attributed to compost addition alone and may reflect the lower N input and resultant N supply relative to U. Two mechanisms may contribute to this positive outcome. First, the lower urea-N input may have reduced excessive plant growth and promoted a more favorable allocation of assimilates to reproductive organs [24,25]. Second, the relatively high N uptake observed in CK indicated substantial indigenous soil N supply. Under such conditions, reduced urea-N may have better synchronized N availability with crop demand and promoted a more favorable source–sink balance [21,23].

In contrast to RU, RU+OF showed higher total N uptake and ANRE but a lower grain yield. Although grain N uptake was significantly higher under RU+OF, NHI did not differ significantly between RU and RU+OF, indicating that the lower grain yield cannot be simply attributed to reduced-N allocation to grain. Together with the lower grain number per panicle and harvest index, these results suggest that the additional N acquired under RU+OF was associated mainly with greater vegetative growth rather than a proportional increase in reproductive sink capacity.

This response may be associated with a mismatch between the N-release pattern of cyanobacterial-sludge-amended compost and reproductive N demand. Continued N availability during later growth may favor vegetative growth without proportionally increasing panicle sink capacity. Previous studies have shown that excessive or poorly synchronized N supply can inhibit grain filling by affecting hormonal regulation and starch synthesis [26,27], while source–sink relationships also influence grain N concentration and dry-matter accumulation [28].

In conclusion, N reduction coupled with compost application did not hinder rice growth. Meanwhile, this practice also provides a potential pathway for the resource utilization of cyanobacterial sludge as compost. RU treatment exhibited the greatest grain yield and harvest index, whereas RU+OF showed greater N uptake, ANRE, and lower NH_3_ volatilization. These findings indicate that greater N uptake does not necessarily translate into higher grain yield unless vegetative growth and reproductive sink development are well coordinated. Therefore, optimizing the substitution ratio of cyanobacterial-sludge-amended compost may help better synchronize nutrient release with grain formation.

### 3.2. Responses of NH_3_ Volatilization to N Reduction Combined with Compost Application

In paddy systems, NH_3_ volatilization is a major N loss pathway [29]. NH_3_ volatilization mainly occurred during the basal and tillering fertilization stages in the present study, which is consistent with previous studies [30]. This was mainly attributed to the high N input rate during these two stages and the limited N uptake by rice seedlings, which increased floodwater NH_4_^+^-N concentration and consequently enhanced NH_3_ volatilization. Similarly, Lin et al. [31] found that floodwater NH_4_^+^-N concentrations and NH_3_ volatilization generally peaked within 1–3 days after N application and increased with the N application rate. Although all treatments showed similar temporal dynamics of NH_3_ volatilization, N management regimes significantly affected the flux [31]. The N reduction treatments generally exhibited lower NH_3_ volatilization rates than the full N treatment, with RU+OF treatment showing the lowest rate. This reduction was more directly associated with the lower urea-N input and reduced short-term accumulation of NH_4_^+^-N in floodwater than with improved crop ANRE. Reducing urea-N decreased the amount of rapidly hydrolysable N entering the floodwater, whereas compost-derived N was likely released more gradually.

Partial substitution of inorganic N with organic fertilizer can reduce cumulative NH_3_ volatilization, mainly by decreasing floodwater NH_4_^+^-N concentrations. However, the magnitude of this effect depends on the substitution rate, water management, and physicochemical properties of the organic fertilizer [32,33]. When chemical fertilizers are applied alone, fertilizer N dissolves quickly and is hydrolyzed by soil urease into NH_4_^+^-N. While part of NH_4_^+^-N is adsorbed by soil colloids, the remainder enters the soil solution, sharply increasing the NH_4_^+^-N concentration and providing sufficient substrate for NH_3_ volatilization [33]. Because RU and RU+OF received the same urea-N input but differed in compost application, the lower NH_3_ volatilization under RU+OF suggests that compost addition may have further modified short-term N availability and NH_4_^+^-N dynamics. This finding is consistent with previous studies identifying floodwater NH_4_^+^-N concentration as a major direct driver of NH_3_ volatilization [34] and showing that organic fertilizers produced using different materials or composting processes can differ substantially in their effects on NH_3_ emissions [35]. However, the specific contribution of compost to reduced NH_3_ volatilization remains uncertain because compost-derived N mineralization was not separately quantified.

In summary, RU treatment achieved the greatest grain yield, whereas RU+OF treatment provided stronger mitigation of NH_3_ volatilization together with greater N uptake and ANRE. This trade-off indicates that compost evaluation should consider not only total nutrient content but also nutrient-release dynamics and their synchronization with crop demand to balance grain yield, N-use efficiency, and environmental emission. Based on these findings, future research should compare multiple compost rates and assess the effects of higher N reduction levels on rice yield, as well as determine whether adjusting application timing can retain the environmental advantages of cyanobacterial-sludge-amended compost while improving reproductive biomass allocation and grain yield. Meanwhile, since these results were based on a pot experiment, field verification is still required.

## 4. Materials and Methods

### 4.1. Background Information and Soil

The experiment was conducted in a greenhouse at Jiangsu Academy of Agricultural Sciences (118.87° E, 32.03° N). The soil from each layer (0–10 cm, 10–30 cm, and 30–50 cm) was carefully repacked into PVC soil columns (60.0 cm in height, 30.0 cm in diameter, with sealed bottoms).

The pot experiment standardized soil, water, fertilizer, and management conditions and facilitated repeated monitoring of floodwater N dynamics and NH_3_ volatilization. It was designed to compare relative treatment responses under controlled conditions rather than reproduce field conditions.

The tested soil was sampled from a reclaimed coastal paddy field under a rice-wheat rotation in Dafeng, Yancheng, Jiangsu Province, China. The experimental site originated from historically saline coastal land that had undergone long-term agricultural reclamation. The selected properties of 0–10 cm topsoil were as follows: pH 7.32 (soil:water, 1:5), organic matter content 15.5 g kg^−1^, total N 0.62 g kg^−1^, available phosphorus 4.52 mg kg^−1^, available potassium 137.5 mg kg^−1^, cation exchange capacity (CEC) 2.64 cmol^+^ kg^−1^ and a sand-silt-clay ratio of contents 22.7%:21.7%:55.6%, respectively.

The cyanobacterial sludge used for compost production was collected from the Taihu Lake region, China. Chicken manure was purchased from Musson Fertilizer, and struvite was prepared in the laboratory by the MAP crystallization method. Details of the composting procedure were reported in our previous study [17]. Some physicochemical properties of the organic fertilizer were moisture content 23%, pH 8.2, organic matter 41.6%, total N 25.5 g kg^−1^, total P 16.7 g kg^−1^, total K 24.7 g kg^−1^, and total nutrients (N + P_2_O_5_ + K_2_O) 9.4%. The rice cultivar used was Nanjing 46. The chemical fertilizers used included analytical-grade urea (AR, 99% purity; Shanghai Yuanye Bio-Technology Co., Ltd., Shanghai, China), with a nominal N concentration of 46.2%, calcium superphosphate (P_2_O_5_ 12%) and potassium chloride (K_2_O 60%).

### 4.2. Experimental Treatments and Managements

Four treatments were established: no urea-N as control (CK), urea-N (240 kg N ha^−1^, U), 80% urea-N (192 kg N ha^−1^, RU), and 80% urea-N plus compost (RU+OF). The organic fertilizer (30% cyanobacterial sludge substituted for chicken manure) was applied at 500 kg·ha^−1^. Each treatment had three replicates. The N and compost application rates were selected based on local farmer practices and previous experiments [36,37]. The treatments represented integrated fertilization regimes; therefore, the individual and interactive effects of N rate, compost application, and compost type were not statistically separated.

Rice seedlings were transplanted on 16 June 2025, with three holes per pot and two seedlings per hole. The total urea-N application was 240 kg N ha^−1^, and was applied as basal (30%), tillering (30%) and panicle (40%) fertilizers on 18 June, 3 July, and 11 August, respectively. For each treatment, 96 kg P_2_O_5_ ha^−1^ and 192 kg K_2_O ha^−1^ were applied as basal fertilizer in forms of calcium superphosphate and potassium chloride. Detailed N, P, K, and OF application rates into each pot were presented in Table 3. Before rice transplanting, the compost was evenly mixed with the 0–10 cm plow layer soil according to the corresponding treatments. The soil was pre-irrigated one week in advance to maintain a flooded condition. Midseason drainage was conducted from 22 to 28 July and rice was harvested on 31 October 2025. All other management practices, including weed and pest control, followed the local conventional farming practices.

### 4.3. Sampling and Measurement

#### 4.3.1. NH_3_ Volatilization Measurement

Daily NH_3_ volatilization fluxes were measured using the continuous power pumping-confined chamber method [38]. A transparent Plexiglas chamber with 15 cm inner diameter and 20 cm height was used for each column. Volatilized NH_3_ was measured by trapping NH_3_ in an absorbing solution (80 mL of 2% boric acid solution mixed with 20 mL methyl red-bromocresol green indicator added per liter). Collection immediately begun on the first day after each N fertilizer application and continued for eight consecutive days, with daily sampling conducted from 8:00 to 10:00 a.m. and from 13:00 to 15:00 p.m. The NH_3_ trapped in the absorbing solution was titrated immediately after collection with 0.02 M H_2_SO_4_ to determine the daily flux. The cumulative NH_3_ volatilization was calculated as the sum of daily values throughout the monitoring period.

#### 4.3.2. pH Value, NH_4_^+^-N, and NO_3_^−^-N Concentration of Floodwater and Soil

Soil samples were collected from the 0–10 cm surface layer using a three-point sampling method on the 8th day after fertilization and after rice harvest. Each sample was divided into two portions: one was air-dried for analysis, and the other was stored at 4 °C. Floodwater samples were collected on the 2nd, 4th, 6th and 8th day after each fertilization. A 50 mL part of floodwater was filtered, transported immediately to the laboratory, and stored frozen in a refrigerator. In situ pH value of the floodwater was measured with a portable pH/mV/conductivity meter (SX72, Shanghai Sanxin Instrument Factory Co., Ltd., Shanghai, China). NO_3_^−^-N and NH_4_^+^-N were determined by colorimetric methods: NO_3_^−^-N using a UV spectrophotometer, and NH_4_^+^-N by the indophenol blue colorimetric method (T-6M, Nanjing Feile Instrument Co., Ltd., Nanjing, China).

#### 4.3.3. Plant Sampling

Plant height and SPAD were measured during the reproductive stage of rice [39]. When the rice matured, the aboveground straw and grains were harvested from each soil column. The number of panicles and grains per panicle were recorded, and straw and grain weights were measured using a balance. To determine dry matter content, 10 randomly selected plants were separated into straw and grains. The samples were first oven-dried at 105 °C for 30 min to deactivate enzymes, followed by drying at 75 °C until constant weight was achieved. The dried samples were ground to pass through a 2 mm sieve. To determine nutrient content, the ground samples were digested with H_2_SO_4_-H_2_O_2_ mixed solution. Total N concentration was determined by the Kjeldahl method (Distillation Apparatus, KD200, Jiasheng Technology Co., Ltd., Hefei, China), and total phosphorus concentration was determined by the molybdenum-antimony spectrophotometric method (T-6M, Nanjing Feile Instrument Co., Ltd., Nanjing, China).

#### 4.3.4. Calculation of N-Use Indices

Apparent nitrogen recovery efficiency (ANRE) and nitrogen harvest index (NHI) were calculated as follows:

$$\mathrm{ANRE}\;\left(\%\right)\;=\;\frac{N\;\mathrm{uptake}\;\mathrm{in}\;N\;\mathrm{application}\;\mathrm{treatment}\;\left(g\right)-\;N\;\mathrm{uptake}\;\mathrm{in}\;\mathrm{control}\;\left(g\right)}{N\;\mathrm{application}\;\mathrm{rate}\;\left(g\right)}\;\times \;100\%$$

$$N\mathrm{HI}\;\left(\%\right)=\frac{N\;\mathrm{uptake},\;\mathrm{grain}}{N\;\mathrm{uptake},\;\mathrm{total}}\;\times \;100\%$$

### 4.4. Data Statistical Analysis

The data were organized using Microsoft Excel 2010 (Microsoft Corporation, Redmond, WA, USA), and figures were plotted using Origin 2024 (OriginLab Corporation, Northampton, MA, USA). Each soil column constituted one independent experimental replicate, and each treatment included three independently managed soil columns (*n* = 3). The data were subjected to analysis of variance, data for each sampling date were analyzed separately using one-way ANOVA to determine the significance of differences among treatments. Results are presented as means ± standard deviations. Duncan multiple comparison tests were conducted to separate means (*p* < 0.05) among treatments (SPSS 21.0 for Windows, SPSS Inc., Chicago, IL, USA).

## 5. Conclusions

We conducted a pot experiment to evaluate the effects of 20% N reduction, alone or in combination with a compost of sawdust and chicken manure plus cyanobacterial sludge on rice yield, ANRE and NH_3_ loss from reclaimed coastal paddy soil. The RU treatment achieved the highest grain yield, with a higher harvest index, more grains per panicle, and higher ANRE than U. Meanwhile, compared with the conventional U treatment, RU+OF treatment showed a higher grain yield, but its greater biomass and N uptake did not result in a higher grain yield than the RU treatment. Interestingly, both RU and RU+OF reduced the NH_3_ volatilization from reclaimed coastal paddy soil, with RU+OF showing the greatest reduction (25.3%). In addition, the 20% N reduction combined with compost resulted in the lowest NH_4_^+^-N and NO_3_^−^-N contents in the overlying water. Overall, moderate N reduction maintained or improved rice yield, while compost addition was associated with greater apparent N recovery and lower NH_3_ volatilization. However, the specific contribution of compost to the reduced NH_3_ volatilization remains uncertain. The compost application rate and substitution ratio require further optimization.

## Figures and Tables

**Table 1:** Rice yield and yield-related agronomic traits under different fertilization treatments.

Treatment	Straw Biomass (g pot^−1^)	Grain Yield (g pot^−1^)	Aboveground Biomass (g pot^−1^)	Panicle Number per Pot	Grains per Panicle	1000-Grain Weight (g)	Harvest Index (%)
CK	85.6 ± 2.4 c	59.3 ± 3.0 d	144.9 ± 4.4 d	19.7 ± 2.1 b	128.0 ± 11.8 ab	26.2 ± 0.3	40.9 ± 0.5 b
U	154.5 ± 12.4 c	87.9 ± 9.0 c	243.5 ± 8.6 c	37.0 ± 2.7 a	110.5 ± 1.7 b	27.3 ± 0.8	36.5 ± 4.1 b
RU	129.3 ± 18.1 b	133.7 ± 5.6 a	270.0 ± 17.0 b	40.0 ± 5.6 a	146.6 ± 15.7 a	28.5 ± 5.6	51.0 ± 3.2 a
RU+OF	193.7 ± 3.1 a	114.1 ± 13.1 b	307.8 ± 13.2 a	40.3 ± 2.5 a	112.8 ± 6.0 b	28.6 ± 1.9	37.0 ± 2.4 b

Note: The pot for experiment was 60 cm in height and 30 cm in diameter. Values are presented as means ± SD (*n* = 3). Different lowercase letters within the same column indicate significant differences among treatments at *p* < 0.05; values without letters were not significantly different among treatments.

**Table 2:** Effects of different fertilization treatments on rice N uptake, apparent N recovery efficiency (ANRE), and N harvest index (NHI).

Treatment	N Uptake (g pot^−1^)			ANRE (%)	NHI (%)
	Straw	Grain	Total		
CK	0.7 ± 0.0 c	0.4 ± 0.1 b	1.1 ± 0.1 c	/	37.9 ± 6.00 a
U	1.1 ± 0.2 ab	0.6 ± 0.2 b	1.7 ± 0.4 b	34.7 ± 0.2 c	35.0 ± 11.0 a
RU	0.9 ± 0.1 bc	0.9 ± 0.0 b	1.8 ± 0.2 b	50.9 ± 0.1 b	52.4 ± 2.00 a
RU+OF	1.2 ± 0.1 a	1.2 ± 0.3 a	2.3 ± 0.2 a	91.4 ± 0.1 a	49.4 ± 10.0 a

Note: The pot for experiment was 60 cm in height and 30 cm in diameter. The values are means ± SD (*n* = 3). Different lowercases letters mean statistically significant difference at *p* < 0.05.

**Table 3:** Fertilizer application rates (g pot^−1^) for different treatments during the rice season.

Treatment	Basal Application				Tillering-Stage Application	Panicle-Stage Application
	Urea	Superphosphate	Potassium Chloride	Compost Product	Urea	Urea
CK	0	4.8	2.2	0	0	0
U	1.1	4.8	2.2	0	1.1	1.5
RU	0.7	3.4	2.2	0	0.7	0.9
RU+OF	0.7	3.4	2.2	53.0	0.7	0.9

## Data Availability

The original contributions presented in this study are included in the article. Further inquiries can be directed to the corresponding author.
